# Pediatric Hip Dysplasia Surgery Outcomes by Pediatric Versus Nonpediatric Orthopedists

**DOI:** 10.7759/cureus.55951

**Published:** 2024-03-11

**Authors:** Sarah Dance, Theodore Quan, Philip M Parel, Benjamin J Farley, Sean Tabaie

**Affiliations:** 1 Orthopaedic Surgery, Children’s National Hospital, Washington, DC, USA; 2 Orthopaedic Surgery, George Washington University School of Medicine and Health Sciences, Washington, DC, USA; 3 Orthopaedic Surgery, Children's National Hospital, Washington, DC, USA

**Keywords:** developmental dysplasia of the hip, hip surgery, nonpediatric orthopedic surgery, pediatric orthopedic surgery, developmental dysplasia of the hip (ddh)

## Abstract

Objectives

Developmental dysplasia of the hip (DDH) encompasses a spectrum of abnormalities in the immature hip. Surgical intervention is indicated if conservative management fails. Despite the increased supply of pediatric orthopedic surgeons (POSs) over the last few decades, there continues to be a maldistribution of surgeons. The purpose of this study is to determine outcomes following surgical management of hip dysplasia by POSs compared to non-pediatric orthopedic surgeons.

Methods

Pediatric patients who underwent surgical treatment for hip dysplasia from 2012 to 2019 were identified using a large national database. Patient demographics, comorbidities, and postoperative complications were compared by pediatric versus nonpediatric-trained orthopedic surgeons. Bivariate and multivariable regression analyses were performed.

Results

Of the 10,780 pediatric patients who underwent hip dysplasia surgery, 10,206 patients (94.7%) were operated on by a POS, whereas 574 (5.3%) were operated on by a non-pediatric orthopedic surgeon. POSs were more likely to operate on patients with a higher American Society of Anesthesiologists class (p<0.001) and those with a greater number of medical comorbidities, including cardiac (p=0.001), gastrointestinal (p=0.017), and neurological (p<0.001). Following analysis using multivariable regression models to control for patient baseline characteristics, there were no differences in any postoperative complications between patients treated by pediatric-trained and nonpediatric-trained orthopedic surgeons.

Conclusions

Compared to non-pediatric orthopedic surgeons, POSs were more likely to operate on younger patients with increased medical comorbidities. However, there were no differences in postoperative complications following surgical management for DDH in patients treated by nonpediatric and pediatric orthopedic surgeons.

## Introduction

Developmental dysplasia of the hip (DDH) encompasses a wide spectrum of abnormalities in the immature hip and is a common condition in the pediatric population, with some articles reporting a prevalence of DDH as high as 1 in every 100 infants [1]. Chronic hip dislocation may result in anatomic changes that alter the range of motion and kinematics of the hip, increase the difficulty of maintaining hip reduction, and contribute to osteoarthritis and avascular necrosis [2,3]. While many cases can be managed nonoperatively, surgical intervention is indicated if conservative management fails or hip dysplasia progresses. Surgical DDH treatment typically is performed by pediatric-trained orthopedic surgeons (POSs).

Despite the increased supply of POSs over the last few decades, there continues to be a maldistribution of POSs across the United States (US), with most clustering in areas of dense population [4,5]. Because of this, there are several geographic areas across the US that lack access to POSs, necessitating nonpediatric-trained orthopedic surgeons (NPOSs) to surgically manage DDH patients. Previous literature has noted improved surgical outcomes in children who underwent surgery by subspecialized pediatric surgeons versus nonpediatric ones [6,7]. However, this relationship has not been explored regarding DDH management. Given the differences in training and focus between POSs and NPOSs, it is imperative to explore whether pediatric subspecialization results in better surgical outcomes. Therefore, the purpose of this study was to determine outcomes following surgical management of hip dysplasia by POSs compared to NPOSs.

## Materials and methods

For this retrospective study from 2012 to 2019, the American College of Surgeons National Surgical Quality Improvement Program-Pediatric (ACS NSQIP-P) database was used, which is a nationally validated database with over 150 participating hospitals and institutions. Trained clinical nurses collect various preoperative and postoperative patient data and enter them into the database. While inaccurate coding and data input may also introduce bias and affect the validity of this study, regular auditing and previous studies have demonstrated high interrater reliability when using the NSQIP database [8]. All patient information in NSQIP-P is de-identified, and this database has been used to analyze outcomes following various surgical procedures [9,10].

Current procedural terminology codes 27140, 27146, 27147, 27151, 27156, 27165, 27258, and 27259 were used to identify all patients who underwent surgical treatment for hip dysplasia, which is consistent with prior studies [11]. To capture only pediatric patients, patients 18 years of age or above were excluded from the study. Two patient groups were categorized for this study: patients who were treated by POSs and patients treated by NPOSs. In NSQIP-P, the surgeon’s specialty can be found for each patient case. Cases with the surgeon’s specialty noted as “pediatric orthopedic surgeon” were classified as having the procedure being performed by a pediatric subspeciality-trained orthopedic surgeon, whereas the cases where the surgeon’s specialty was noted as “orthopedic surgeon” were considered to be performed by a non-pediatric subspeciality-trained orthopedic surgeon. This methodology is consistent with prior studies [12].

Baseline characteristics

Various patient characteristics were collected from the database. Demographics and clinical/surgical characteristics included gender, race, age, American Society of Anesthesiologists (ASA) classification, and total operative time (time from surgical incision to closure). Comorbidities were also collected and were grouped into categories, including cardiac (any cardiac risk factors, previous cardiac surgery, cardiopulmonary resuscitation within seven days of surgery, or inotropic support at the time of surgery), pulmonary (asthma, oxygen support, tracheostomy, chronic lung disease, structural pulmonary/airway abnormalities, or ventilator dependence), renal (renal insufficiency or renal failure), gastrointestinal (esophageal, gastric, or intestinal disease), biliary (biliary, liver, or pancreatic disease), and neurological (structural central nervous system abnormality, seizure disorder, developmental delay, cerebral palsy, or neuromuscular disorder). Immune disease, steroid use within 30 days of surgery, nutritional support requirement, recent weight loss or failure to thrive, hematologic or bleeding disorder, and bone marrow or solid organ transplant were also collected.

Postoperative complications

Thirty-day complications assessed included superficial/deep surgical site infections, wound dehiscence, urinary tract infection, pneumonia, unplanned reintubation, renal failure, seizure, peripheral nerve injury, cardiac arrest, bleeding requiring transfusion, venous thromboembolism, Clostridium difficile infection, sepsis, extended length of hospital stay, readmission, reoperation, and mortality. Extended length of stay was defined as more than seven days or one standard deviation above the mean length of stay of 3.5 days for the patients in this study.

Statistical analysis

To compare the differences in baseline characteristics and postoperative complications between the two groups of patients, Pearson’s Chi-squared test and analysis of variance were used where appropriate. Demographic and comorbidity variables with a p-value <0.20 were included in the multivariable regression analysis to control for confounders [13]. Multivariable regression results were reported as odds ratios with 95% confidence intervals. A p-value <0.05 was statistically significant, and all analyses were done using IBM SPSS Statistics for Windows, Version 28 (Released 2021; IBM Corp., Armonk, New York, United States).

## Results

Demographics and clinical characteristics

A total of 10,780 pediatric patients underwent hip dysplasia surgery and were included in the study. Ten thousand two hundred and six patients (94.7%) were operated on by a POS, whereas 574 (5.3%) were operated on by a non-pediatric subspecialty-trained orthopedic surgeon. Compared to NPOSs, POSs were more likely to operate on younger patients (p<0.001) and patients with a higher ASA class (p<0.001). POSs also had a longer operative time when performing surgical procedures compared to NPOSs (p=0.004) (Table 1).

**Table 1:** Demographics and Clinical Characteristics Among Hip Dysplasia Surgical Patients ^¶^Pearson’s chi-squared test **Analysis of variance Bolding equals significance p<0.05 ASA, American Society of Anesthesiologists; SD, standard deviation.

Variables	Pediatric Surgeons	Non-pediatric Surgeons	p-value
Total patients, n	10,206	574	
Sex, n (%)			0.155^¶^
Female	5,665 (55.5)	336 (58.5)	
Male	4,541 (44.5)	238 (41.5)	
Race, n (%)			0.070^¶^
White	5,669 (63.9)	343 (63.2)	
Black or African American	1,253 (14.1)	96 (17.7)	
Hispanic	1,576 (17.8)	79 (14.5)	
American Indian or Alaska Native	40 (0.5)	1 (0.2)	
Asian	297 (3.3)	23 (4.2)	
Native Hawaiian or Pacific Islander	35 (0.4)	1 (0.2)	
ASA, n (%)			< 0.001^¶^
I	1,410 (17.5)	119 (26.4)	
II	3,125 (38.9)	173 (38.4)	
III	3,394 (42.2)	150 (33.3)	
IV	108 (1.3)	8 (1.8)	
Mean age, yrs (SD)	8.23 (4.80)	9.63 (5.30)	< 0.001**
Mean operation time, mins (SD)	204.38 (96.23)	192.61 (99.24)	0.004**

Comorbidities

Relative to NPOSs, pediatric-trained surgeons were more likely to operate on patients with a greater number of comorbidities, including cardiac (p=0.001), gastrointestinal (p=0.017), and neurological (p<0.001), as well as those who required nutritional support (p=0.015) (Table 2).

**Table 2:** Comorbidities Among Hip Dysplasia Surgical Patients ^¶^Pearson’s chi-squared test Bolding equals significance p<0.05

Comorbidities	Pediatric Surgeons	Non-pediatric Surgeons	p-value^ ¶^
Total patients, n	10,206	574	
Cardiac comorbidity, n (%)	1,256 (12.3)	45 (7.8)	0.001
Pulmonary comorbidity, n (%)	1,902 (18.6)	93 (16.2)	0.144
Renal comorbidity, n (%)	5 (0.2)	0 (0.0)	0.654
Gastrointestinal comorbidity, n (%)	1,917 (18.8)	85 (14.8)	0.017
Biliary comorbidity, n (%)	12 (0.5)	0 (0.0)	0.487
Neurological comorbidity, n (%)	5,855 (57.4)	287 (50.0)	< 0.001
Immune disease, n (%)	17 (0.7)	0 (0.0)	0.408
Steroid use, n (%)	134 (1.3)	6 (1.0)	0.582
Nutritional support, n (%)	1,792 (17.6)	78 (13.6)	0.015
Failure to thrive, n (%)	64 (3.3)	1 (1.2)	0.277
Hematologic disorder, n (%)	276 (2.7)	17 (3.0)	0.712
Bleeding disorder, n (%)	20 (0.8)	0 (0.0)	0.369
Bone marrow transplant, n (%)	9 (0.4)	0 (0.0)	0.548
Solid organ transplant, n (%)	7 (0.3)	0 (0.0)	0.596

Complications

On bivariate analysis, when compared to patients operated on by POSs, patients who were operated on by NPOSs were more likely to experience cardiac arrest within 30 days of surgery (p=0.013) (Table 3). Following adjustment on multivariable regression analysis to control for potential confounders, which included the comorbidities, there were no differences in any postoperative complications between patients treated by pediatric-trained and non-pediatric orthopedic surgeons. 

**Table 3:** Bivariate Analysis of Postoperative Complications of Hip Dysplasia Surgical Patients ^¶^Pearson’s chi-squared test Bolding equals significance p<0.05

Complications	Pediatric Surgeons	Non-pediatric Surgeons	p-value^¶^
Total patients, n	10,206	574	
Superficial surgical site infection, n (%)	74 (0.7)	4 (0.7)	0.938
Deep surgical site infection, n (%)	26 (0.3)	1 (0.2)	0.707
Organ/space infection, n (%)	10 (0.1)	1 (0.2)	0.578
Superficial wound dehiscence, n (%)	61 (1.1)	4 (1.1)	0.917
Deep wound dehiscence, n (%)	13 (0.1)	0 (0.0)	0.392
Urinary tract infection, n (%)	98 (1.0)	3 (0.5)	0.290
Pneumonia, n (%)	108 (1.1)	4 (0.7)	0.406
Reintubation, n (%)	54 (0.5)	3 (0.5)	0.983
Renal failure, n (%)	2 (0.0)	0 (0.0)	0.737
Seizure, n (%)	6 (0.1)	1 (0.2)	0.291
Nerve injury, n (%)	13 (0.1)	1 (0.2)	0.762
Cardiac arrest, n (%)	6 (0.1)	2 (0.3)	0.013
Postoperative transfusion, n (%)	2,012 (19.7)	128 (22.3)	0.131
Venous thromboembolism, n (%)	13 (0.1)	0 (0.0)	0.392
Clostridium difficile infection, n (%)	18 (0.2)	0 (0.0)	0.292
Sepsis, n (%)	51 (0.5)	1 (0.2)	0.273
Extended length of stay (> 7 days), n (%)	566 (5.6)	22 (3.8)	0.076
Readmission, n (%)	510 (8.3)	26 (6.8)	0.308
Reoperation, n (%)	258 (4.3)	20 (5.3)	0.333
Mortality, n (%)	6 (0.1)	1 (0.2)	0.292

## Discussion

Increasing subspecialization, greater restrictions on duty hours and supervision in the operating room, and the rapid growth of orthopedic knowledge have caused a demand for orthopedic subspecialty training [14]. Despite this, all board-certified orthopedic surgeons in the US are required to prove competency in each orthopedic subspecialty during their five-year residency. Given the increase in subspecialization, recent literature has been interested in determining any differences in undergoing surgery with specialized versus non-specialized surgeons. This is the first study comparing postoperative outcomes following DDH procedures between pediatric- and nonpediatric-trained orthopedic surgeons utilizing a large, national database. The study reveals that POSs are more likely to operate on younger patients with more comorbidities compared to their nonpediatric-trained counterparts. However, after adjusting for potential confounders, the difference in postsurgical complication rates between POSs and NPOSs was nonsignificant.

It is of little surprise that pediatric-trained orthopedic surgeons are more likely to operate on a pediatric population that is younger, has increased medical comorbidities, and has higher ASA scores compared to NPOSs. In addition to their expertise in treating pediatric patients, POSs are likely to operate at hospitals with resources dedicated to a large pediatric population, where anesthesiologists and clinical support staff are also well-versed in pediatric physiology and managing pediatric comorbidities and potential emergencies. In contrast, NPOSs may not have access to these same resources and are more likely to refer medically complex patients to a medical center better equipped at handling any potential complications.

Perhaps more surprising is the result from our study indicating significantly longer operative time among POSs compared to NPOSs. This finding may relate to what Seibold et al. hypothesized in their study comparing POS and NPOS postsurgical outcomes following femoral shaft fixation in a pediatric population, in which the authors concluded that the increased medical complexity of patients treated by pediatric-trained specialists may require different surgical approaches or increased peri-operative stabilization that contribute to longer operation times [7]. Looking at differences in pediatric and general hospitals' surgical management of adolescent idiopathic scoliosis, Russell et al. found no statistically significant difference in procedural time, although they did note a thirty-minute delay of procedural start time at the pediatric hospital due to mandated procedural timeouts conducted in the preoperative holding area [15]. Unfortunately, our study design did not allow us to stratify for potential reasons for prolonged operative times. It is possible that the types of procedures being performed by POSs and NPOSs differ, resulting in longer operative times in the POS group compared to the NPOS one.

Our study also explored the thirty-day complication rate following surgical DDH treatment. On bivariate analysis, patients undergoing DDH surgical treatment by NPOS were at increased risk of cardiac arrest compared to patients managed by POS, although this difference was negligible when controlling for potential confounders with multivariate analysis. Articles reviewing the postoperative differences between pediatric- and nonpediatric-trained orthopedic surgeons following non-DDH surgeries have yielded similar results. Although researching femoral shaft fracture fixation, Seibold et al. found minimal differences in complications between each cohort, with prolonged hospital stay being the only complication at increased risk after treatment by NPOS [7]. Jenkins et al. and Farley et al. also found no difference in postoperative outcomes between both cohorts, concluding that supracondylar humeral fracture fixation can be safely performed by pediatric and nonpediatric orthopedic surgeons alike [16,17]. Interestingly, when reviewing the relationship between surgeon experience in pediatric surgeries across multiple surgical specialties, including orthopedic surgery, neurosurgery, otolaryngology, and cardiothoracic surgery, Rhee et al. reported that the improved mortality outcomes seen following general and cardiothoracic surgeries by pediatric-trained surgeons did not extend to the field of orthopedic surgery [6]. Even in adult studies, the effects of surgeon type and training on surgical outcomes remain unclear [18]. A study by Silva et al., however, demonstrated how the increased pediatric surgical experience was found to significantly reduce the need for open reduction and percutaneous pinning in the treatment of supracondylar humerus fractures, but they note that multiple factors likely influence this assertion [19].

While our results allude to minimal differences in surgical outcomes in the DDH population, this study is not without its limits. First, the methodology of the study relies on CPT codes to identify patients, which, while they are used frequently to identify a particular research population, their primary purpose is for billing. Additionally, CPT codes are not necessarily specific to a diagnosis of developmental hip dysplasia, which may present some inherent bias in the results. The database does not differentiate by type of surgical procedure, which may also affect the results. Furthermore, the ACS NSQIP-P database only follows surgical outcomes thirty days after surgery, meaning that deductions of long-term patient outcomes and complication differences cannot be made from our research. It is important to note that the majority of reoperations and mechanical complications occur more than thirty days from the initial surgery within the field of orthopedics [20]. This study also neglects to consider that NPOSs that operate frequently within the pediatric population may have better outcomes than their more adult-focused counterparts when performing surgery for DDH. Finally, some bias may exist due to the significant disparity in size between the POS and NPOS cohorts. Future studies should consider monitoring postoperative outcomes for a longer follow-up period in a population identified by both CPT and International Classification of Diseases 9/10 (ICD-9, ICD-10) codes to ensure appropriate diagnosis and treatment. In addition, reviewing whether surgical technique changes between POS and NPOS would be helpful in determining the relationship between fellowship training and patient outcomes in orthopedic surgery. Finally, future studies could better stratify each cohort based on the type of procedures performed and severity of DDH.

## Conclusions

Despite the rise in POSs, certain geographic areas across the United States have limited access to one, necessitating treatment of pediatric orthopedic conditions, like DDH, by NPOSs. The results indicated that, compared to nonpediatric-trained orthopedic surgeons, POSs were more likely to operate on more difficult patients with increased medical comorbidities. While the results show that there are minimal differences in postoperative complications between both cohorts, it is important to note the limitations in our study, such as lack of stratification by procedure type and time to diagnosis of DDH, which can significantly affect patient outcomes. 
